# A Case of Symptomatic Intraluminal Internal Carotid Artery Thrombus in a Patient with Essential Thrombocythemia Surgically Treated by CEA

**DOI:** 10.1155/2023/9152009

**Published:** 2023-11-24

**Authors:** Satoshi Takahashi, Masahiro Katsumata, Hirotsugu Nogawa, Kento Takahara, Jin Nakahara, Masahiro Toda

**Affiliations:** ^1^Department of Neurosurgery, Keio University, School of Medicine, Tokyo, Japan; ^2^Department of Neurology, Keio University, School of Medicine, Tokyo, Japan

## Abstract

We report a patient with a symptomatic intraluminal internal carotid artery thrombus clinically revealed by cerebral infarction. In the preoperative evaluation, it was revealed that essential thrombocythemia existed in the background. Therefore, medical treatment with antithrombotic agents in conjunction with hydroxycarbamide for essential thrombocythemia was initiated, but the thrombus was not dissolved by three weeks. At this time, the patient underwent carotid endarterectomy, which removed the thrombus completely with its adjacent plaque without any perioperative stroke. The possibility of essential thrombocythemia may also be kept in mind when an increased platelet count is observed in patients with internal carotid artery thrombus. It is a reasonable option to precede medical treatment, including anticoagulant therapy, by setting the time limit for surgical intervention in such a disease state.

## 1. Introduction

Intraluminal internal carotid artery (ICA) thrombus is a disease classically thought to be urgent and requiring surgical extraction. However, there are also occasional reports that medical treatment should be prioritized due to the high rate of embolic complications at the time of surgery. Its course of treatment remains controversial [1, 2].

With regard to symptomatic carotid artery stenosis resulting in stroke, approximately 70% of patients with 70–99% stenosis on medical therapy have been reported to have no subsequent recurrence of cerebral infarction for 5 years with the best medical treatment [3]. On the other hand, according to the Cochrane Review updated in 2017, CEA is reported to be highly beneficial for symptomatic carotid stenosis with 70–99% stenosis [4].

Essential thrombocythemia (ET) is a myeloproliferative disorder in which the platelet count increases. Its clinical manifestations can be complicated by opposing haemorrhagic and thrombotic complications. Arterial and venous thrombosis is reported to occur in 14% of cases at the time of diagnosis [5].

Regarding the relationship between ET and intraluminal ICA thrombosis, as far as we know, only five cases of intraluminal ICA thrombus in patients with ET have been reported so far, and only one out of five cases was surgically treated [6–10].

In this paper, we report on a patient with intraluminal ICA thrombus with a background of ET. We also discuss the treatment strategy and the timing of surgical intervention for patients like this one, including a review of the literature.

## 2. Case Presentation

**Figure 1:**
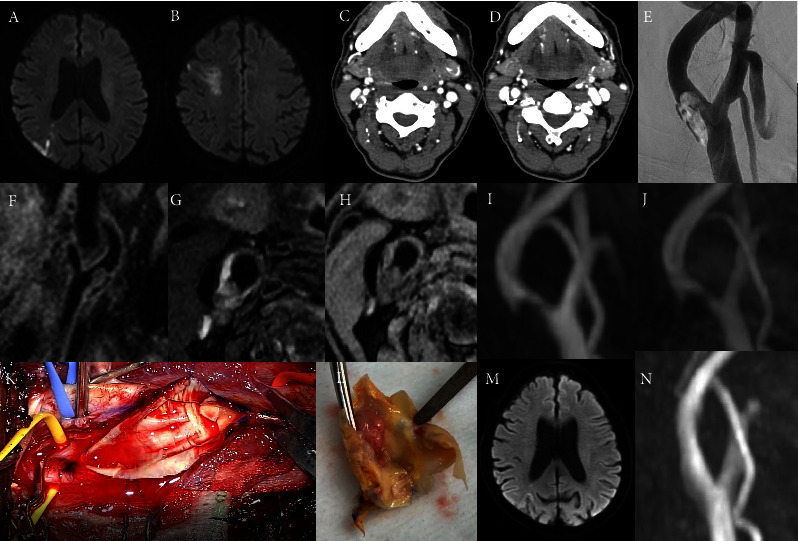
Imaging Findings. (A, B) Magnetic resonance diffusion-weighted images obtained at admission. Scattered subacute-phase cerebral infarction in the right hemisphere. (C, D) 3D computed tomography angiography showed high-grade stenosis of the right internal carotid artery. (E) A translucent lesion is recognized in the right internal carotid artery on cerebral angiography and diagnosed as an internal carotid artery floating thrombus. (F–H) Carotid plaque image. Sagittal (F) T2 axial. (G) T1 position. (H) T1 shortening is absent, suggesting that time has elapsed since thrombus formation. (I, J) Initial cervical magnetic resonance angiography (MRA) after admission (I) and cervical MRA after three weeks of antithrombotic therapy (J). (K) Intraoperative carotid endarterectomy (CEA) findings. It did not differ from conventional CEA except for an impression of slightly more bleeding. (L) Carotid artery internal thrombus removed per plaque. A white thrombus is also observed. (M, N) Magnetic resonance imaging as seen 2 days after surgery. No obvious new cerebral infarction was observed (M), and carotid artery stenosis was confirmed to have been released (N).

The patient was an Asian man in his 70 s with no remarkable medical history who had mild paralysis of the left upper extremity for one and a half months before his referral to our institution, for which he was diagnosed with spinal canal stenosis and treated in the nearby orthopaedic clinic. Since dysarthria was also recognized in progress, the patient was referred to our institution. Our examination found scattered subacute-phase cerebral infarction in the right cerebral hemisphere on MRI (Figures 1(A) and 1(B)). The signal intensity of diffusion-weighted imaging was variable, suggesting that it may be a cerebral infarction in the subacute phase that progressed in a stepwise manner. No apparent ischemic changes in the deep white matter were observed in the left hemisphere, and subacute cerebral infarction was confined only to the right hemisphere. On admission, he was aware of dysarthria that was not obvious objectively, and mild motor palsy with a slightly positive Barre's sign was observed in the left upper extremity. The NIHSS score on admission was 2, and the modified Rankin Scale of the patient was 1. Hemoglobin A1c was within normal limits, and no dyslipidemia was observed in blood sampling. An echocardiogram showed no remarkable abnormal findings, and his cardiac function was diagnosed as normal by a cardiologist. Dual antiplatelet therapy (DAPT) consisting of 100 mg of low-dose aspirin in combination with 200 mg of cilostazol, continuous intravenous infusion of 40 mg/day argatroban, and 5 mg of rosuvastatin calcium was started, and CT angiography performed based on a causal search revealed high-grade stenosis of the right ICA (Figures 1(C) and 1(D)). The translucent appearance of the ICA defect was recognized on cerebral angiography, which was carried out to judge the necessity of surgical intervention, and an intraluminal ICA thrombus was diagnosed (Figure 1(E)). A NASCET 80% stenosis was revealed by the cerebral angiography. MRI plaque imaging was also carried out (Figures 1(F)–1(I)). The carotid plaque was T1 isointense, T2 high intense, and TOF low intense to the adjacent muscles that was not typical findings for fragile plaque. After the intraluminal ICA thrombus was diagnosed, his cilostazol was changed to clopidogrel 75 mg, argatroban was changed to heparin, and his target APTT was adjusted to 40–50 seconds. Since the platelet count increased to 505 × 10^3^ per *μ*l on admission and 783 × 10^3^ per *μ*l after admission, the possibility of ET was suspected, and the patient was seen by the haematology department. As a result of bone marrow aspiration, hyperplasia of the bone marrow was recognized, the diagnosis of ET with JAK2 mutation electropositivity was confirmed by genetic analysis, and hydroxycarbamide treatment was started.

Concerning the intraluminal ICA thrombus, since there was a background of ET and the possibility of growing the thrombus again after the extraction, even in the CAS or CEA, and obvious mobility was not recognized in the thrombus on carotid artery echo, it was decided to prioritize medical treatment, including heparin continuous administration first, and to carry out CEA if the disappearance of the thrombus was not observed. The CAS was judged to be difficult from the size of the thrombus, so it was decided to carry out thrombus retrieval by endovascular treatment only in the case of large vessel occlusion caused by distal embolus from thrombus detachment.

Three weeks after starting antithrombotic therapy, no new neurological deficit symptoms were observed, and no new cerebral infarction was observed on imaging, but MRA and carotid artery echo showed no resolution of the thrombus (Figure 1(J)). The stenosis rate of the carotid artery remained at about NASCET 80% without remarkable changes from the time of the initial cerebral angiography. Since the disappearance of the thrombus could not be expected from the continuation of medical treatment, including heparin, and we were concerned that long-term continuation of heparin would raise the risk of haemorrhagic complications, it was decided to perform CEA. Clopidogrel was discontinued 3 days before surgery, and low-dose aspirin was given as the single antiplatelet agent. Heparin was stopped 6 hours before the operation.

Although this CEA was performed mostly as usual, systemic heparinization was performed when the common carotid artery and the external carotid artery were secured (heparinization was usually performed after exposure of all vessels, including the ICA), the ICA was dissected more gently, and the distal ICA was secured only after confirming the prolongation of ACT. After the ICA was closed, it remained closed until removal of the thrombus was completed. When doing CEA, we selectively inserted the shunt for patients without ischemic tolerance, and no shunt was used in this case (Figure 1(K)). The thrombus was white and firm and appeared compatible with white thrombus, protruding into the internal carotid artery with the plaque as a scaffold (Figure 1(L)). The connection between the plaque and the thrombus was rigid and not in a readily detached context. The adhesion of the white thrombus in a way that would raise concerns about dispersion was not recognized at the surface of the thrombus.

**Figure 2:**
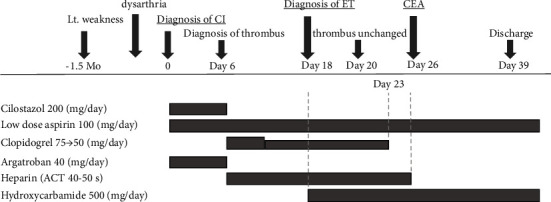
Course of treatment. Lt.: left, CI: cerebral infarction, ET: essential thrombocythemia, CEA: carotid endarterectomy, and ACT: activated clotting time.

The postoperative course was uneventful, and MR imaging the following day also showed relieved stenosis with no new artifacts (Figures 1(M) and 1(N)). The treatment for ET was also considered important, so low-dose aspirin and hydroxycarbamide were continued in the perioperative period. No troubles, such as skin ulcers that have been reported as a side effect of hydroxycarbamide, were recognized. No obvious new neurological sequelae were recognized on the 13th postoperative day, and the patient was discharged home for independent walking. No recurrence of the intraluminal ICA thrombus has been recognized, even in the recent ambulatory treatment 22 months after the surgery. The clinical course of the patient is summarized in Figure 2.

## 3. Discussion

We have detailed the treatment of a patient in whom ET was first diagnosed at the time of an in-depth evaluation of cerebral infarction. ET is a rare condition with an incidence of several persons per million in Japan, while the incidence of thrombosis in patients with ET is as high as 14% [5, 11]. Thrombosis most commonly affects small- and medium-sized vessels, and thrombosis of large vessels rarely occurs; however, some aortic and carotid thrombi related to ET have been previously reported [12]. The thrombotic property of ET was considered in this patient's symptomatic intraluminal ICA thrombus.

As far as we have found, five cases of intraluminal extradural ICA thrombus in patients with ET have been reported in the literature so far (summarized in Table 1) [6–10]. Including the present case, five of the six cases were located in the cervical ICA near the bifurcation where carotid plaque often develops, and the other case was located in the petrous portion of the ICA [9]. It was reported that the intraluminal thrombus formed a plaque as a scaffold [10], and we think this is the reason intraluminal ICA thrombi in patients with ET are often found in the cervical ICA near the bifurcation. Four of the six cases were treated conservatively with medical treatment, and thrombi disappeared without causing additional infarction in three of the four. In the fourth case, the thrombus grew, and the ICA was totally occluded by the thrombus, causing massive cerebral infarction despite the patient being put on antiplatelet therapy [7]. Huh et al. reported a case of symptomatic carotid stenosis treated by emergent CEA [6]. In that case, intra-arterial thrombus occurred 1 POD after emergent CEA, and ET was diagnosed by subsequent workup. In that case, the only antiplatelet therapy administered before the initial surgery for symptomatic carotid stenosis was clopidogrel. Taken together, the evidence suggests anticoagulant therapy is important for preventing thrombi developing. Another important point from the case reported by Huh et al. was the possibility that the thrombus of patients with ET would be exacerbated by the wound caused by direct operation on the carotid artery [6]. Fortunately, the recurrence of the thrombus in our patient has not been recognized, even in the latest examination one and a half year after the operation.

There have been some reports on the resolution of intraluminal ICA thrombi by medical treatment with antithrombotic drugs alone [8–10]; however, in this case, the thrombus was not decreased after three weeks of medical treatment, including DAPT plus argatroban followed by heparin. In our institution, we treat cases with atherothrombotic cerebral infarction in which the extensive cerebral infarction has not been completed by DAPT plus argatroban. Since continuous administration of argatroban has only been permitted for up to 48 hours under the insurance system in Japan, argatroban has been changed to heparin as in this case, if it is judged necessary to continue anticoagulation therapy for a longer period of time. The POINT trial showed that in patients with ischemic stroke, those who received a combination of clopidogrel and low-dose aspirin had a lower risk of major ischemic events but a higher risk of major haemorrhage than those who received aspirin alone [13]. In this regard, it may be worrisome that antithrombotic therapy with DAPT plus argatroban is too potent. On the other hand, it has been reported that DAPT and argatroban can be safely used for cerebral infarction in mild to moderate with improved neurological outcome [14, 15].

The symptom of cerebral infarction was first thought to be caused by cervical spondylosis, so antithrombotic therapy was started only approximately 1.5 months after the onset of cerebral infarction. The internal carotid artery thrombus identified intraoperatively was firm, and we thought the lack of resolution of the thrombus by medical treatment was due to the time lag between the formation of the thrombus and the initiation of antithrombotic therapy. However, since the new cerebral infarction was not recognized after the start of antithrombotic therapy, we consider that a therapeutic plan that surgically treats a case only after medical treatment does not lead the disappearance of the thrombus in a few weeks is also reasonable for intraluminal ICA thrombi diagnosed even in the subacute stage. We consider that preceding medical treatment contributed to stabilizing the intraluminal thrombus. In the meantime, since the haemorrhagic complication of antithrombotic therapy is also a concern, the timing of surgical intervention should be planned from the beginning to be resorted to when no disappearance tendency is recognized in the thrombus, even if the antithrombotic therapy has been given.

Regarding the selection of a surgical strategy between CEA and CAS, we thought it would be difficult to retrieve all thrombi without any distal dispersion in CAS due to the size of the thrombus. There was also a concern of plaque shift into the stent, so CEA was performed. The patient was concerned about haemorrhagic and ischemic complications after surgery because of his background of ET. The surgeon had the impression that the intraoperative bleeding tendency was greater than that of usual CEA. Since DAPT was performed up to 3 days before the operation, the effect of antiplatelet drugs was also considered, but the possibility that the patient showed a bleeding tendency due to ET could not be ruled out. Careful haemostasis was performed at the time of wound closure, and no haemorrhagic complications were observed postoperatively. There was also no relapse of the thrombus that was of concern preoperatively. It has also been reported that the intraluminal thrombus formed the plaque in the scaffold [10], and endarterectomy was carried out simultaneously in the operation, which was preferable from the viewpoint of preventing thrombus relapse. In this case, although the plaque that became the scaffold of the thrombus was not thick enough to cause advanced stenosis of the internal carotid artery, it seemed to be important to extract the plaque because it could become the scaffold of the thrombus. In this regard, we consider that CEA is better than CAS when the extraction of the plaque is not possible.

## 4. Conclusions

We report on a patient with intraluminal ICA thrombus with a background of ET. The possibility of ET may also be kept in mind when an increased platelet count is observed in patients with intraluminal ICA thrombus. A literature review in combination with our experience from the present case suggested that antithrombotic therapy, especially anticoagulant therapy, is important for preventing the progression of intraluminal thrombi in such cases. Since intraluminal ICA thrombosis is reported to often disappear by medical treatment only, it is a reasonable option to start with medical treatment, including anticoagulant therapy, and to see if this dissolves the thrombus within a set time limit, after which the option of surgical intervention can be executed.

## Figures and Tables

**Table 1:** Summary of six cases with intraluminal ICA thrombus in patients with ET.

	Location	Treatment	Outcome	Surgery
Huh et al. [6]	Cervical ICA	Emergency CEA before ET diagnosis	Intraluminal ICA thrombus occurred 1POD after emergent CEA	Yes

Kato et al. [7]	Cervical ICA	Antiplatelet	ICA occlusion	No

Vassileva et al. [8]	Cervical ICA	Antiplatelet	Improved	No

Vemmos et al. [9]	Petrous potion	Anticoagulant	Improved	No

Yamada et al. [10]	Cervical ICA	Antiplatelet+ Anticoagulant	Improved No additional infarction	No

Present case	Cervical ICA	Antiplatelet+ Anticoagulant CEA	Improved No additional infarction	Yes

## Data Availability

The data used to support the findings of this study are included within the article.

## References

[1] Bhatti A. F., Leon L. R., Labropoulos N. (2007). Free-floating thrombus of the carotid artery: literature review and case reports. *Journal of Vascular Surgery*.

[2] Combe J., Poinsard P., Besancenot J. (1990). Free-floating thrombus of the extracranial internal carotid artery. *Annals of Vascular Surgery*.

[3] Naylor A. R., Sillesen H., Schroeder T. V. (2015). Clinical and imaging features associated with an increased risk of early and late stroke in patients with symptomatic carotid disease. *European Journal of Vascular and Endovascular Surgery*.

[4] Rerkasem A., Orrapin S., Howard D. P., Rerkasem K. (2020). Carotid endarterectomy for symptomatic carotid stenosis. *Cochrane Database of Systematic Reviews*.

[5] Tefferi A., Pardanani A. (2019). Essential thrombocythemia. *New England Journal of Medicine*.

[6] Huh I. Y., Han I. S., Lee H. K., Shin Y. J., Lee J. M. (2018). Recurrent thrombosis after carotid endarterectomy secondary to activated protein C resistance and essential thrombocytosis: a case report. *Medicine (Baltimore)*.

[7] Kato Y., Nagamine Y., Hayashi T., Takao M. (2018). Extending carotid artery thrombus associated with thrombocytosis. *Internal Medicine*.

[8] Vassileva E., Daskalov M., Stamenova P. (2015). Free-floating thrombus in stroke patients with nonstenotic internal carotid artery-an ultrasonographic study. *Journal of Clinical Ultrasound*.

[9] Vemmos K. N., Spengos K., Tsivgoulis G., Manios E. (2004). Progressive stroke due to essential thrombocythemia. *European Journal of Internal Medicine*.

[10] Yamada K. Y., Yoshimura S., Yamakawa H., Iwama T. (2008). Cerebral infarction associated with mobile plaque in a patient with essential thrombocythemia. *Journal of Neuroendovascular Therapy*.

[11] Suzuki K., Sezai A., Tanaka M. (2020). Unsuccessful surgical treatment of thoracic aortic thrombosis in a patient with essential thrombocythemia. *Journal of Cardiac Surgery*.

[12] Geringer J., Fenderson J., Osswald M. (2019). Essential thrombocythemia complicated by occlusive thrombosis of the abdominal aorta. *Case Reports in Hematology*.

[13] Johnston S. C., Easton J. D., Farrant M. (2018). Clopidogrel and aspirin in acute ischemic stroke and high-risk TIA. *New England Journal of Medicine*.

[14] Li X. Q., Hou X. W., Cui Y. (2022). Safety and preliminary efficacy of argatroban plus dual antiplatelet therapy for acute mild to moderate ischemic stroke with large artery atherosclerosis. *Brain and Behavior*.

[15] Zhou L. S., Li X. Q., Zhou Z. H., Chen H. S. (2020). Effect of argatroban combined with dual antiplatelet therapy on early neurological deterioration in acute minor posterior circulation ischemic stroke. *Clinical and Applied Thrombosis*.

